# Regulation of appressorium development in pathogenic fungi

**DOI:** 10.1016/j.pbi.2015.05.013

**Published:** 2015-08

**Authors:** Lauren S Ryder, Nicholas J Talbot

**Affiliations:** School of Biosciences, University of Exeter, Stocker Road, Exeter EX4 4QD, United Kingdom

## Abstract

Many plant pathogenic fungi have the capacity to breach the intact cuticles of their plant hosts using specialised infection cells called appressoria. These cells exert physical force to rupture the plant surface, or deploy enzymes in a focused way to digest the cuticle and plant cell wall. They also provide the means by which focal secretion of effectors occurs at the point of plant infection. Development of appressoria is linked to re-modelling of the actin cytoskeleton, mediated by septin GTPases, and rapid cell wall differentiation. These processes are regulated by perception of plant cell surface components, and starvation stress, but also linked to cell cycle checkpoints that control the overall progression of infection-related development.

## Highlights

• Appressorium development is linked to cell cycle checkpoints controlling morphogenesis.
• Ras GTPase signalling acts upstream of cAMP and MAP kinase pathways for appressorium development.
• Melanin is not exclusively associated with appressorium turgor generation.
• .
• Septin-mediated actin re-modelling is essential for appressorium function.
• Focal secretion of effectors occurs during appressorium infection.

**Current Opinion in Plant Biology** 2015, **26**:8–13

This review comes from a themed issue on **Biotic interactions**

Edited by **Uta Paszkowski** and **D Barry Scott**

For a complete overview see the Issue and the Editorial

Available online 1st June 2015

**http://dx.doi.org/10.1016/j.pbi.2015.05.013**

1369-5266/© 2016 The Authors. Published by Elsevier Ltd. This is an open access article under the CC BY license (http://creativecommons.org/licenses/by/4.0/).

## Introduction

Plant pathogenic fungi cause many of the world's most devastating crop diseases and each year significant expenditure is required to combat plant diseases and thereby ensure food security [1, 2]. The problem is even more pressing in the developing world, where the high cost of fungicides means that disease outbreaks have serious consequences; farmers frequently face economic ruin and the societal and economic impact of plant diseases is significant. It has been estimated that up to 30% of the global harvest is lost each year to plant disease and therefore identifying durable solutions to plant diseases is likely to be one of the most important means by which plant productivity can be increased in a sustainable way [2, 3].

**Figure 1:**
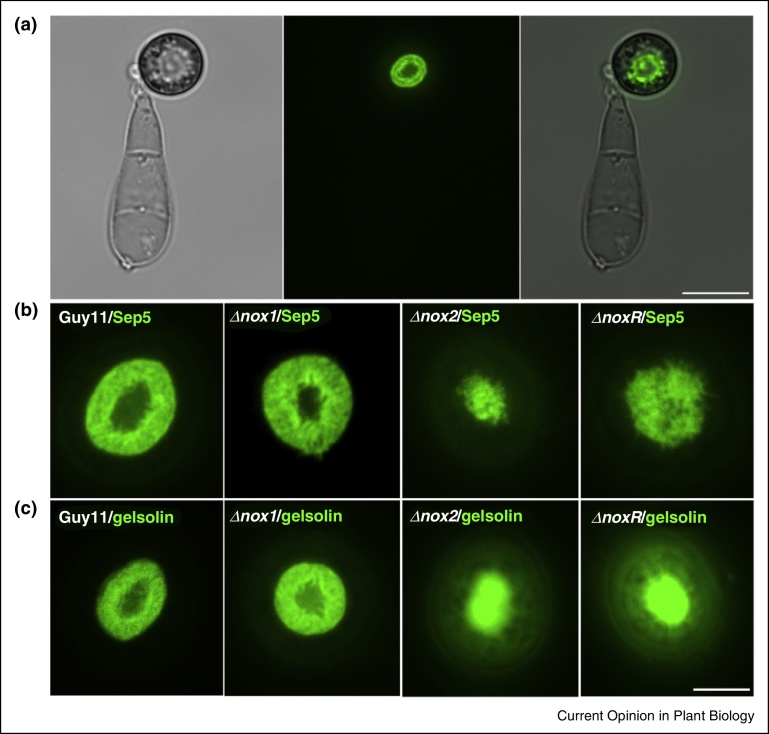
Photomicrographs showing appressorium development by the rice blast fungus *Magnaporthe oryzae*. Conidia were inoculated onto hydrophobic glass coverslips and incubated in a moist chamber at 26 °C for 8 hours. **(a)** Bright field, epifluorescence and merged images to show localization of the Sep5-GFP septin gene fusion in a hetero-oligomeric ring at the base of the appressorium. The septin ring is necessary for re-modelling F-actin to the appressorium pore [29]. Bar = 10 μm. **(b)** Septin localization to the appressorium pore is dependent on regulated synthesis of ROS by the Nox2 NADPH oxidase and its regulatory NoxR sub-unit. Sep5-GFP localization in a *Δnox1, Δnox2* and *ΔnoxR* mutant. **(c)** Nox2-dependent localization of the actin-binding protein gelsolin. Gelsolin-GFP localization in a *Δnox1, Δnox2* and *ΔnoxR* mutant. See [32^•^] for details. Bar for (b) and (c) = 5 μm.

Many plant pathogenic fungi have evolved the capacity to breach the intact cuticles of their plant hosts by elaborating specialised structures called appressoria [4, 5]. These cells can take various forms — either single-celled structures, or compound appressoria composed of numerous cells, which can collectively form structures known as infection cushions [6]. In many cases appressoria are simple terminal swellings at the tips of germ tubes that emerge from spores on the leaf surface [7], whereas in other species such as the rice blast fungus, *Magnaporthe oryzae* and the anthracnose disease-causing *Colletotrichum* species, appressoria are melanin-pigmented, septate structures that initially form at the tips of germ tubes, but then differentiate into dome-shaped fully differentiated infection structures [7] (see Figure 1).

In this review, we compare and evaluate recent studies that have investigated the biology of appressorium development in plant pathogenic fungi. Many of the studies focus on model plant pathogenic species, such as the rice blast fungus *M. oryzae* and the corn smut fungus *Ustilago maydis* [8]. These two species are diverse — *M. oryzae* is an ascomycete and *U. maydis* a basidiomycete. However, there are some common themes emerging from studies of both of these species, and indeed among other appressorium-forming fungi. There is, for example, an emerging picture of a highly orchestrated developmental process requiring perception of physical and chemical cues from the plant leaf surface, coupled with control of both nuclear and cell division. Targeting such fundamental morphogenetic processes may therefore be important in terms of developing the next generation of anti-penetrant fungicides, or targeting plant-based methods to control some of the most important cereal diseases [3, 4].

## Early appressorium development

Early appressorium development occurs soon after a spore lands on the surface of its host. In the rice blast fungus *M. oryzae* a three-celled conidium germinates within an hour of attaching itself to the leaf surface which it does by means of an adhesive, specially adapted to adhere tightly to the hydrophobic, waxy leaf cuticle [4]. Upon hydration and surface contact, the spore rapidly germinates and sends out a germ tube, normally emerging from the tapering end of the three-celled conidium. The germ tube extends for 10–15 μm before flattening at its tip, hooking, and beginning to differentiate into the unicellular appressorium. Control of initiation of appressorium development is based on perception of hydrophobicity (the surface needs to have water contact angles of greater than 90 degrees, typical of plastic surfaces such as Teflon) and surface hardness [3]. In addition, the fungus is able to respond to wax monomers such as 1,16-hexadecanediol, which are powerful inducers of appressorium development at the leaf surface [3]. However, in addition to the perception of physical cues, cell cycle control is pivotal to development appressoria [9]. Each compartment of the three-celled conidium contains a single nucleus and the cell from which the germ tube emerges undergoes a single round of nuclear division, before appressorium development [9]. Entry of this conidial nucleus into DNA replication (S-phase) is necessary for initiation of appressorium development [10] and inhibiting DNA replication, either with hydroxyurea or by generation of a temperature-sensitive nim1 mutant, which undergoes aberrant mitosis in the absence of DNA replication, completely prevents the ability of germ tubes to differentiate at their tips [10]. Subsequently, appressorium maturation and melanisation is controlled by entry of the nucleus into G2 and mitosis. Only if mitosis occurs does the appressorium become fully functional. At this point cytokinesis occurs and a contractile actomyosin ring forms at the neck of the appressorium, which differentiates the cell from the rest of the pre-penetration structures [11]. Autophagy is then stimulated within the conidium, such that all of the intracellular contents of the three-celled spore are degraded before being trafficked to the incipient appressorium [9]. The culmination of this process is turgor generation within the appressorium of up to 6–8 MPa, which is sufficient to breach the underlying rice cuticle [4, 12]. Blocking autophagy by targeted mutation of any of the 16 genetic components of the non-selective macroautophagy pathway is sufficient to render the fungus non-pathogenic [13]. Interestingly, cell cycle control of appressorium development is likely to be a conserved process [10]. In *U*. *maydis*, for example, cell cycle arrest is necessary for an infective filament of the fungus to be able to penetrate plant tissue [14^••^]. *U. maydis* undergoes a self-/non-self-recognition process on the corn leaf surface in which two monokaryotic sporidia fuse to form an infectious dikaryotic filament [8]. This forms an appressorium, which is necessary to breach the corn leaf surface [14••, 15]. Recent evidence suggests that cell cycle arrest is required for plant infection. The cell cycle arrest results by cooperation of at least two distinct underlying mechanisms, one of these involves activation of the DNA damage response cascade, and the other relies on transcriptional regulation of a gene called *HSL1*, which encodes a protein kinase that modulates the G2 to M transition [14••, 15]. Thus, the control of nuclear division and its coordination with morphogenesis at the leaf surface appear to be processes which are fundamental to penetration of the cuticle by diverse plant pathogens [8]. Appressorium formation also relies on perception of physical and biochemical cues at the leaf surface. It has long been recognized that in *M. oryzae* appressorium morphogenesis involves the Pmk1 MAP kinase pathway and the cAMP response pathway, but how these pathways interact has not been clear [16, 17]. Recent evidence has suggested that the Mac1 adenylate cyclase interacts with Cap1, a cyclase-associated protein that activates adenylate cyclase and is potentially involved in re-modelling the actin cytoskeleton with which it appears to strongly associate based on its localization pattern in appressoria [18^•^]. In *M. oryzae*, the Pmk1 MAPK pathway is necessary for appressorium development [16]. Upstream of Pmk1 a number of potential receptors are involved in perception of surface signals [3]. *PTH11*, for example, a CFEM domain G-protein coupled receptor, is necessary for perception of the hydrophobic leaf surface by *M. oryzae* and in its absence, appressoria do not form [19]. RAS signalling is likely to act upstream of the Pmk1 and cAMP response pathways because generation of a dominant-active allele of Ras2 (*RAS2*^*G17V*^) leads to abnormal appressorium formation in the absence of a surface, such that appressorium-like structures can be formed by aerial hyphae [20^••^]. Expression of the dominant *M. oryzae RAS2*^*G17V*^ allele in *Colletotrichum graminicola* and *C. gloeosporioides* also led to aerial appressoria suggesting conservation of the surface perception signalling mechanism [20^••^]. The Pmk1 kinase cascade is composed of three protein kinases, Mst11, Mst7 and Pmk1, which appear to be scaffolded by a protein called Mst50, which interacts with Mst11, and upon activation and phosphorylation of its components, a phosphor-relay is triggered leading to the detachment of Pmk1 and its traversal to the nucleus during appressorium maturation [21, 22]. Recent transcriptional profiling results and interaction studies suggest that several transcription factors operate downstream of Pmk1, including Mst12 and Mcm1, which likely activate a large set of gene products involved in cell wall differentiation, and the physiological changes associated with appressorium maturation, turgor generation, in addition to the control of autophagy and programmed cell death of the conidium that precedes appressorium maturation [23]. Interestingly, the Pmk1 pathway also appears to regulate microconidia formation by *M. oryzae*, because Pmk1 and Mst12 mutants show reduced microconidia production while Mcm1 is essential for their development. Microconidia may represent an alternative means of propagation by the pathogen to facilitate rapid spread within plant tissue [24^••^].

The Pmk1 pathway is widely conserved in other plant pathogenic fungi and is likely to be required for infection-related morphogenesis, although the diversity of these processes in different plant pathogens and the absence of systemic comparative analysis, has precluded detailed analysis [8].

## Appressorium turgor generation

Maturation of the appressorium in *M. oryzae* is accompanied by rapid synthesis of glycerol and other polyols, leading to turgor generation and formation of a thick differentiated melanin layer on the inner side of the appressorium cell wall, which is required to retard efflux of glycerol from the rapidly expanding appressorium and also to provide structural rigidity and resilience to the infection cell [4, 12]. Interestingly, it has long been held that melanin in the appressorium serves a role to maintain turgor pressure due to lowering the porosity of the appressorium cell wall. However, recent experiments have shown that in the anthracnose pathogen of corn, *Colletotrichum graminicola*, turgor accumulates even when melanin biosynthesis is inhibited and the penetration of intact leaves and artificial substrates still occurs [25^•^]. Moreover, cell collapse assays (cytorrhysis) analysis of the appressorial osmolyte content using a method called Mach-Zehnder interferometry, showed that melanin is not required for solute accumulation and turgor generation [25^•^]. This suggests that melanin may not provide the barrier for osmolytes in *C. graminicola*, in the way it does in *M. oryzae* [25•, 26]. Instead, it seems likely that melanin plays a structural role because albino mutants, lacking the CgPKS1 polyketide synthase gene involved in 1,3,6,8-tetrahydroxy-naphthalene biosynthesis, were prone to rupture and impaired in their ability to cause disease [25^•^]. Experiments with the soybean rust fungus, *Phakopsora pachyrhizi* demonstrated that high turgor, of up to 5.13 MPa, could be observed in its non-melanised appressoria [27, 28••]. This analysis was carried out using transmitted light double-beam interference Mach-Zehnder microscopy. The study highlights how hyaline (non-pigmented) appressoria of rust fungi, such as *P. pachyrhizi*, can generate turgor in the absence of melanin in their cell walls [28^••^]. Turgor generation still requires accumulation of osmotically active polyols, but these can apparently be retained even in the absence of melanin. Clearly, therefore cell walls of appressoria must have evolved in different ways to maintain turgor, some of which do not require melanin. Although there is a clear role for melanin in structural rigidity and turgor generation in fungi such as *C. graminicola* and *M. oryzae* [25•, 26], it may not serve the same function, while other non-melanised fungi may still undertake mechanical appressorium-mediated infection [27, 28••].

## Appressorium maturation and cuticle rupture

Recent experiments have begun to address how appressoria change their axis of polarity and re-establish polarised growth at the interface between the fungus and the plant [29]. This is necessary to focus turgor in the appressorium, associated with isotropic expansion of the cell, into physical force at the base of the infection cell, leading to generation and protrusion of the penetration peg into the cuticle [29].

The appressorium pore defines the point at the base of the infection cell from which the penetration hypha emerges. In *M. oryzae* and *Colletotrichum* species, the appressorium pore is clearly distinct from the rest of the infection cell, with a much thinner cell wall and the absence of melanin. This is visible by ultra-structural analysis [29, 30]. The appressorium pore is the site of remodelling of the actin cytoskeleton [29, 30, 31]. During penetration peg formation rapid F-actin polymerisation occurs at this point leading to rapid polarised growth of the penetration hypha. Re-modelling of actin requires morphogenetic septin GTPases [29, 32•]. A septin ring of approximately 5.9 μm was observed at the appressorium pore of *M. oryzae* and is composed of four core septins, Sep3, Sep4, Sep5 and Sep6. The septin ring is necessary for scaffolding actin, leading to formation of a toroidal F-actin network at the base of the appressorium [29]. The septin ring also acts as a lateral diffusion barrier, tethering in place proteins implicated in F-actin polymerisation, such as the Las17 component of the arp2/3 complex. In addition, ezrin, radixin, moesin (ERM) domain proteins required for actin membrane interactions at the cortex of cells, were found to be located within the septin ring at the appressorium pore, in addition to BAR domain proteins implicated in the control of membrane curvature generation [29]. Eukaryotic cells undergo membrane curvature generation in order to generate invaginations associated, for instance, with endocytosis and also cellular protrusions, such as lamellipodia found in epithelial cells [33]. Such cellular protrusions require membrane curvature to be stimulated, followed by rapid membrane biogenesis and F-actin polymerisation. These processes must be spatially regulated to the point of plant infection and this appears to be one of the key roles that septins play during the control of appressorium polarisation in *M. oryzae* [29]. Recent evidence has suggested that a reactive oxygen species burst catalysed by the Nox2 NADPH oxidase is necessary for septin-mediated appressorium re-polarisation [32^•^]. Nox2 and its regulatory subunit NoxR are required for septin ring formation at the base of the appressorium and a second NAPDH oxidase, encoded by the *NOX1* gene, is necessary for maintenance of the polarised growth and organisation of the toroidal F-actin network at the base of the appressorium during penetration peg formation [32^•^]. Mutation of genes encoding any of the septin components and either of the *NOX2* and *NOXR* genes is sufficient to prevent plant infection and, indeed, the appressorium pore fails to differentiate from the rest of the infection cell. By contrast, mutation of *NOX1* leads to arrest of the penetration process just after differentiation of a stunted penetration peg, which fails to elongate and breach the cuticle [32•, 34]. Reactive oxygen species (ROS) generated within the appressorium may act in at least two different ways to stimulate cytoskeletal re-modelling. ROS may act directly on proteins such as gelsolin, which are involved in actin severing and formation of free barbed ends that stimulate rapid F-actin polymerisation [35]. This prediction is based on experiments in which the action of latrunculin, an actin depolymerising agent, could be competitively inhibited by the presence of ROS in *M. oryzae* appressoria, leading to penetration peg formation [32^•^]. Additionally, ROS probably acts on signalling components that operate downstream of a turgor sensor (or sensors) that must operate in the appressorium to define the point at which re-polarisation needs to be triggered. This is likely to be upstream of the formation of the hetero-oligomeric septin ring. Components involved in this process likely include Chm1, a protein kinase implicated in septin phosphorylation [29, 32•, 36, 37].

## The penetration peg as site of effector delivery

Plant infection by pathogens involves deployment of effector proteins that suppress plant immunity responses and facilitate proliferation of the pathogen within plant tissues [for review see 38^••^]. Ultra-structural analysis of *C. higginsianum* has detected effectors within the appressorium pore at the point of plant infection [30], highlighting how the penetration peg allows rapid deployment of effectors early during the infection process. This is consistent with evidence that specialised focal secretion mechanisms for effectors are likely to be present in both *Colletotrichum orbiculare* [39^••^] and *M. oryzae* [40^•^]. An essential pre-requisite for focal secretion of effectors at the penetration peg and extending primary infection hypha, is a means of communication between the extending hyphal tip and the fungal nucleus, which is still within the appressorium on the leaf surface. In *U. maydis*, a recent study has shown that a retrograde, early endosome-mediated, long-distance signalling pathway is necessary for transcriptional regulation of effector genes and effector secretion from the hyphal tip during plant tissue colonisation [41^••^].

## Future prospects

Recent studies have demonstrated that there is more diversity in the manner in which appressorium turgor is generated than was hitherto appreciated [25•, 26, 27, 28••]. However, some common themes in appressorium morphogenesis have also emerged, such as the importance of cell cycle control and the operation of a widely conserved MAP kinase pathway for appressorium differentiation. Another key advance has been to study for the first time how isotropic expansion of appressoria is translated into the generation of invasive forces necessary to breach the leaf surface [29, 32•]. There is also an emerging picture of the appressorium pore as a key signalling hub during plant infection that is not only the site of rapid fungal growth, but also the site of initial secretion of fungal effectors and associated regulatory components [39••, 40•]. Future experiments will need to define precisely how focal effector secretion is regulated and the likely conservation of early endosome-mediated signalling [41^••^]. Moreover, the precise mechanism by which the appressorium monitors turgor needs to be defined, because this must determine the optimal point for re-polarisation and host cell penetration. Experiments to identify the turgor sensing mechanisms of appressoria are therefore required, as well as an understanding of how this triggers regulated synthesis of ROS and lead to septin-mediated cytoskeletal re-organisation. Rapid progress in generating mutants by high throughput targeted genome editing and silencing, provide a means to identify new components of these regulatory networks. The prospect of utilising the new genome editing techniques, such as CRISPR-Cas9 methodologies [42^••^], in this regard is arguably the most exciting means by which numerous fungal genes could be tested to establish their role in appressorium biology and plant infection.

## References and recommended reading

Papers of particular interest, published within the period of review, have been highlighted as:

• of special interest
•• of outstanding interest

## References

[1] Pennisi E. (2010). Armed and dangerous. Science.

[2] Fisher M.C., Briggs C.J., Brownstein J.S., Madoff L.C., McCraw S.L., Gurr S.J. (2012). Emerging fungal threats to animal, plant and ecosystem health. Nature.

[3] Wilson R.A., Talbot N.J. (2009). Under pressure: investigating the biology of plant infection by *Magnaporthe oryzae*. Nat Rev Microbiol.

[4] Talbot N.J. (2003). On the trail of a cereal killer: exploring the biology of *Magnaporthe grisea*. Annu Rev Microbiol.

[5] Dean R.A., Talbot N.J., Ebbole D.J., Farman M.L., Mitchell T.K., Orbach M.J., Thon M., Kulkarni R., Xu J.R., Pan H. (2005). The genome sequence of the rice blast fungus *Magnaporthe grisea*. Nature.

[6] Armentrout V.N., Downer A.J. (1986). Infection cushion development by *Rhizoctonia solani* on cotton. Phytopathology.

[7] Mendgen K., Hahn M., Deising H. (1996). Morphogenesis and mechanisms of penetration by plant pathogenic fungi. Annu Rev Phytopathol.

[8] Perez-Nadales E., Filomena M., Nogueira A., Baldin C., Castanheira S., Ghalid M.E., Grund E., Lengeler K., Marchegiani E., Mehrotra P.V. (2014). Fungal model systems and the elucidation of pathogenicity determinants. Fungal Genet Biol.

[9] Veneault-Fourrey C., Barooah M., Egan M., Wakley G., Talbot N.J. (2006). Autophagic fungal cell death is necessary for infection by the rice blast fungus. Science.

[10] Saunders G.O., Aves S.J., Talbot N.J. (2010). Cell cycle-mediated regulation of plant infection by the rice blast fungus. Plant Cell.

[11] Saunders G.O., Dagdas Y.F., Talbot N.J. (2010). Spatial uncoupling of mitosis and cytokinesis during appressorium-mediated plant infection by the rice blast fungus *Magnaporthe oryzae*. Plant Cell.

[12] de Jong J.C., McCormack B.J., Smirnoff N., Talbot N.J. (1997). Glycerol generates turgor in rice blast. Nature.

[13] Kershaw M.J., Talbot N.J. (2009). Genome-wide functional analysis reveals that infection-associated fungal autophagy is necessary for rice blast disease. Proc Natl Acad Sci USA.

[14••] Castanheira S., Mielnichuk N., Pérez-Martín J. (2014). Programmed cell cycle arrest is required for infection of corn plants by the fungus *Ustilago maydis*. Development.

[15] Mielnichuk N., Sgarlata C., Perez-Martin J. (2009). A role for the DNA-damage checkpoint kinase Chk1 in the virulence program of the fungus *Ustilago maydis*. J Cell Sci.

[16] Xu J.R., Hamer J.E. (1996). MAP kinase and cAMP signalling regulate infection structure formation and pathogenic growth in the rice blast fungus *Magnaporthe grisea*. Genes Dev.

[17] Adachi K., Hamer J.E. (1998). Divergent cAMP signaling pathways regulate growth and pathogenesis in the rice blast fungus *Magnaporthe grisea*. Plant Cell.

[18•] Zhou X., Zhang H., Li G., Shaw B., Xu J.R. (2012). The cyclase-associated protein Cap1 is important for proper regulation of infection-related morphogenesis in *Magnaporthe oryzae*. PLoS Pathog.

[19] DeZwaan T.M., Carroll A.M., Valent B., Sweigard J.A. (2003). *Magnaporthe grisea* Pth11p is a novel plasma membrane protein that mediates appressorium differentiation in response to inductive substrate cues. Plant Cell.

[20••] Zhou X., Zhao X., Xue C., Dai Y., Xu J.R. (2014). Bypassing both surface attachment and surface recognition requirements for appressorium formation by over active Ras signaling in *Magnaporthe oryzae*. MPMI.

[21] Zhao X.H., Kim Y., Park G., Xu J.R. (2005). A mitogen-activated protein kinase cascade regulating infection-related morphogenesis in *Magnaporthe grisea*. Plant Cell.

[22] Park G., Chaoyang X., Zhao X., Yangseon K., Orbach M., Xu J.R. (2006). Multiple upstream signals converge on an adaptor protein Mst50 to activate the *PMK1* pathway in *Magnaporthe grisea*. Plant Cell.

[23] Zhou X., Liu W., Wang C., Xu Q., Wang Y., Ding S., Xu J.R. (2011). A MADS-box transcription factor MoMcm1 is required for male fertility, microconidium production and virulence in *Magnaporthe oryzae*. Mol Microbiol.

[24••] Zhang H., Wu Z., Wang C., Li Y., Xu J.R. (2014). Germination and infectivity of microconidia in the rice blast fungus *Magnaporthe oryzae*. Nat Commun.

[25•] Ludwig N., Lohrer M., Hempel M., Mathea S., Schliebner I., Menzel M., Kiesow A., Schaffrath U., Deising H.B., Horbach R. (2014). Melanin is not required for turgor generation but enhances cell-wall rigidity in appressoria of the corn pathogen *Colletotrichum graminicola*. MPMI.

[26] Howard R.J., Ferrari M.A., Roach D.H., Money N.P. (1991). Penetration of hard substrates by a fungus employing enormous turgor pressures. Proc Natl Acad Sci USA.

[27] Chang H.X., Miller L.A., Hartman G.L. (2014). Melanin-independent accumulation of turgor pressure in appressoria of *Phakopsora pachyrhizi*. Phytopathology.

[28••] Loehrer M., Botterweck J., Jahnke J., Mahlmann D.M., Gaetgens J., Oldiges M., Horbach R., Deising H., Schaffrath U. (2014). In vivo assessment by Mach-Zehnder double-beam interferometry of the invasive force exerted by the Asian soybean rust fungus (*Phakopsora pachyrhizi*). New Phytol.

[29] Dagdas Y.F., Yoshino K., Dagdas G., Ryder L.S., Bielska E., Steinberg G., Talbot N.J. (2012). Septin-mediated plant cell invasion by the rice blast fungus, *Magnaporthe oryzae*. Science.

[30] Kleemann J., Rincon-Rivera L.J., Takahara H., Neumann U., van Themaat E.V.L., van der Does H.C., Hacquard S., Stüber K., Will I., Schmalenbach W., Schmelzer E., O’Connell R.J. (2012). Sequential delivery of host-induced virulence effectors by appressoria and intracellular hyphae of the phytopathogen *Colletotrichum higginsianum*. PLoS Pathogens.

[31] Bourett T.M., Howard R.J. (1990). *In vitro* development of penetration structures in the rice blast fungus *Magnaporthe grisea*. Can J Bot.

[32•] Ryder L.S., Dagdas Y.F., Mentlak T.A., Kershaw M.J., Thornton C.R., Schuster M., Chen J., Wang Z., Talbot N.J. (2013). NADPH oxidases regulate septin-mediated cytoskeletal remodeling during plant infection by the rice blast fungus. Proc Natl Acad Sci USA.

[33] Zhao H., Pykäläinen A., Lappalainen P. (2011). I-BAR domain proteins: linking actin and plasma membrane dynamics. Curr Opin Cell Biol.

[34] Egan M.J., Wang Z.Y., Jones M.A., Smirnoff N., Talbot N.J. (2007). Generation of reactive oxygen species by fungal NADPH oxidases is required for rice blast disease. Proc Natl Acad Sci USA.

[35] Kwiatkowski D.J. (1999). Functions of gelsolin: motility, signaling, apoptosis, cancer. Curr Opin Cell Biol.

[36] Versele M., Thorner J. (2004). Septin collar formation in budding yeast requires GTP binding and direct phosphorylation by the PAK, Cla4. J Cell Biol.

[37] Li L., Xue C., Bruno K., Nishimura M., Xu J.R. (2004). Two PAK kinase genes, CHM1 and MST20, have distinct functions in *Magnaporthe grisea*. Mol Plant Microbe Interact.

[38••] Giraldo M.C., Valent B. (2013). Filamentous plant pathogen effectors in action. Nat Rev Microbiol.

[39••] Irieda H., Maeda H., Akiyama K., Hagiwara A., Saitoh H., Uemura A., Terauchi R., Takano Y. (2014). *Colletotrichum orbiculare* secretes virulence effectors to a biotrophic interface at the primary hyphal neck via exocytosis couple with SEC22-mediated traffic. Plant Cell.

[40•] Giraldo M.C., Dagdas Y.F., Gupta Y.K., Mentlak T.A., Yi M., Martinez-Rocha A.L., Saitoh H., Terauchi R., Talbot N.J., Valent B. (2013). Two distinct secretion systems facilitate tissue invasion by the rice blast fungus *Magnaporthe oryzae*. Nat Commun.

[41••] Bielska E., Higuchi Y., Schuster M., Steinberg N., Kilaru S., Talbot N.J., Steinberg G. (2014). Long-distance endosome trafficking drives fungal effector production during plant infection. Nat Commun.

[42••] Ran F.A., Hsu P.D., Wright J., Agarwala V., Scott D.A., Zhang F. (2013). Genome engineering using the CRISPR-Cas9 system. Nat Protoc.

